# Homeostatic Model Assessment for Insulin Resistance Is Associated With Late Miscarriage in Non-Dyslipidemic Women Undergoing Fresh IVF/ICSI Embryo Transfer

**DOI:** 10.3389/fendo.2022.880518

**Published:** 2022-06-17

**Authors:** Tianli Yang, Yuanyuan Yang, Qiong Zhang, Donge Liu, Nenghui Liu, Yumei Li, Zhongyuan Yao, Yeqing Zhang, Fen Tian, Jing Zhao, Yanping Li

**Affiliations:** ^1^Reproductive Medicine Center, Xiangya Hospital of Central South University, Changsha, China; ^2^Clinical Research Center for Women’s Reproductive Health in Hunan Province, Changsha, China

**Keywords:** HOMA-IR, insulin resistance, late miscarriage, assisted reproductive technology, IVF/ICSI-ET

## Abstract

**Objective:**

To evaluate the associations between homeostatic model assessment for insulin resistance (HOMA-IR) and pregnancy outcomes in non-dyslipidemic infertile women undergoing *in vitro* fertilization/intracytoplasmic sperm injection-embryo transfer (IVF/ICSI-ET).

**Materials and Methods:**

This is a retrospective study involving 3,615 non-dyslipidemic infertile women who attend to the Reproductive Medicine Center of Xiangya Hospital, Central South University (CSU) between January 2014 and October 2021. Eligible participants were divided into three groups according to the quartiles of HOMA-IR: Group 1 (HOMA-IR <1.46), Group 2 (1.46 to <2.71) and Group 3 (HOMA-IR ≥2.71). Baseline data, clinical characteristics during the assisted reproductive technology (ART) procedure, pregnancy, and neonatal outcomes were compared among the three groups. Subgroup analysis based on presence or absence of the polycystic ovary syndrome (PCOS) status was also performed to analyze the effects of HOMA-IR among non-PCOS populations.

**Results:**

The late miscarriage rate and percentage of macrosomia increased with the HOMA-IR group (for late miscarriage rate: 2.23% vs. 3.04% vs. 7.35%, P<0.001; for macrosomia: 0.21% vs. 1.70% vs. 3.23%, P=0.002). Increased HOMA-IR (HOMA-IR≥2.71) was positively associated with late miscarriage (crude OR 3.50, 95% CI 1.64-7.47, P=0.001; adjusted OR 3.56, 95% CI 1.56-8.15, P=0.003). In the subgroup analysis, there were 3,165 participants in the non-PCOS group and 450 were assigned to the PCOS group. Late miscarriage rate increased with the HOMA-IR group among non-PCOS populations (2.20% vs. 3.03% vs. 7.67%, P<0.001). Late miscarriage rate of PCOS women were comparable among the three HOMA-IR groups (2.50% vs. 3.06% vs. 5.71%, P=0.634). Among non-PCOS women, increased HOMA-IR (HOMA-IR≥2.71) was positively associated with late miscarriage (crude OR 3.71, 95% CI 1.66-8.30, P=0.001; adjusted OR 3.82, 95% CI 1.59-9.17, P=0.003).

**Conclusions:**

Late miscarriage rate and prevalence of macrosomia increased with the HOMA-IR index. Preconception HOMA-IR is an independent risk factor for late miscarriage in normolipidemic women undergoing IVF/ICSI-ET. Controlling insulin resistance before ART might prevent the occurrence of late miscarriage and macrosomia.

## Highlights

Preconception HOMA-IR is an independent risk factor for late miscarriage in normolipidemic women undergoing IVF/ICSI-ET.

## Introduction

Insulin resistance (IR) refers to an impairment in insulin-mediated control of glucose homeostasis, which is characterized by hyperinsulinemia and defective response of target cells or a whole organism to the insulin exposure (1, 2). Any defects in insulin signaling reduce insulin sensitivity in targeted tissues and cells (3). IR associates with a constellation of long-term morbidities including type 2 diabetes mellitus (T2DM) (4), obesity (5), metabolic syndrome (6), cardiovascular disease (7) and cognitive dysfunction (8). Except for these, it is noteworthy that insulin signaling is essential for female fecundity and IR may adversely impact the reproductive functions (9–11).

To date, a plethora of studies with respect to the effects on female fertility favored polycystic ovary syndrome (PCOS) populations for the high prevalence of IR among them (12). IR induces hyperandrogenemia and ovulatory dysfunction by disturbing the hypothalamic-pituitary-ovarian (HPO) axis and exerting a direct effect on ovarian theca cells (13). Hyperinsulinemia increases the amplitude and frequency of gonadotropin releasing hormone (GnRH)-stimulated luteinizing hormone (LH), and an increased LH and the follicle stimulating hormone (FSH) ratio, in turn, impairs downstream ovarian folliculogenesis and alters steroid hormone production towards androgen excess (14). Evidence from IR mouse models indicated that maternal IR contributed oxidative stress and defective mitochondrial function in germinal vesicle (GV) and metaphase II (MII) oocytes, which potentially impaired oocyte quality and early embryonic development (10). Insulin signaling was required for human endometrial decidualization *via* modulating cellular glucose uptake that met the growing energy demands of decidual cells (9). Decreased apoptosis of stromal cells was observed in early pregnant insulin-exposed mice, in which the decidualization process was markedly compromised (11) In light of these observations, hyperinsulinemia and IR are linked to poor fertility.

As the gospel for infertile couples, assisted reproductive technology (ART) enables these couples to obtain pregnancy. However, various obstacles hinder the achievement of this goal and unsatisfactory pregnancy outcomes have been reported among women presenting with IR. In recent meta-analysis, IR and high body mass index (BMI) were two risk factors for spontaneous abortion in infertile PCOS patients who underwent ART (15). Even after adjusting for PCOS status, the homeostatic model assessment for insulin resistance (HOMA-IR) was positively associated with the risk of spontaneous miscarriage during fresh embryo transfer (16). In women with HOMA-IR greater than 4.5, the odds ratio (OR) is eight times that of those with HOMA-IR of 4.5 or less (16). Moreover, other studies reported negative associations between IR and oocyte-embryo quality in the ART scenarios (17, 18).

Previously, the clinical impact of hyperinsulinemia or IR on female reproductive abnormalities was mostly confined to PCOS populations. Even for non-PCOS individuals, the majority of previous research did not take serum lipid levels into consideration. Our recent real-world analysis revealed that dyslipidemia was negatively associated with live birth rate among infertile women underwent their first *in vitro* fertilization/intracytoplasmic sperm injection-embryo transfer (IVF/ICSI-ET) cycle (19). Therefore, to rule out the potential effect of serum lipid levels, this study aims to analyze the association between IR and pregnancy outcomes among non-dyslipidemic infertile women by using a convenient and inexpensive surrogate marker HOMA-IR (20).

## Materials and Methods

### Study Population

This study was a retrospective analysis and approved by the ethical committee of Xiangya Hospital of Central South University (CSU) (No. 2021008). All patients underwent the first IVF/ICSI-ET cycle in the Reproductive Medicine Center of Xiangya Hospital, CSU between January 2014 and October 2021. This study was conducted according to the tenets of the Declaration of Helsinki. Because of its retrospective nature the informed consent of individual patients was waived.

The inclusion criteria were as follows: women aged between 20 and 40 years, normal blood lipids profile according to the Chinese adult dyslipidemia management guideline (21), serum triglyceride (TG) level <1.7mmol/l, total cholesterol (TC) level <5.2mmol/l, low-density lipoprotein cholesterol (LDL-C) level <3.4mmol/l and high-density lipoprotein cholesterol (HDL-C) level ≥1.0mmol/l, and first complete fresh IVF/ICSI cycle with freshly ejaculated semen from patient’s husband and at least one good quality cleavage-stage embryos transferred.

The exclusion criteria were as follows: women with diagnosed diabetes mellitus or had a history of hypoglycemic and hypolipidemic medications within three months before the ART treatment;severe hydrosalpinx and did not receive tubal ligation or salpingectomy; severe adenomyosis; endometrial abnormalities such as endometrial polyps, endometrial hyperplasia, submucosal fibroids, intrauterine adhesions or chronic endometritis without management; genital tuberculosis; other severe systemic comorbidities, such as hypertension, prethrombotic conditions, autoimmune connective tissue diseases and malignant tumor. Moreover, semen or oocytes from donated cycles were also excluded.

Eligible participants were divided into three groups according to the quartiles of HOMA-IR (P25: 1.46, P75: 2.71, see **Supplementary Table 1**): Group 1 (HOMA-IR <1.46), Group 2 (HOMA-IR ≥1.46 and <2.71) and Group 3 (HOMA-IR ≥2.71). Subgroup analysis based on presence or absence of the PCOS status was also performed to analyze the effects of HOMA-IR among non-PCOS populations. PCOS was diagnosed according to the Rotterdam Consensus criteria (2 out of 3) as follows: 1) oligo- or anovulation; 2) clinical and/or biochemical signs of hyperandrogenism; and 3) polycystic ovaries and exclusion of other etiologies (congenital adrenal hyperplasia, androgen-secreting tumors, Cushing’s syndrome).

### Sample Assessment and ART Procedure

Before officially proceeding with the commencement of the IVF/ICSI cycle, the patient’s median cubital venous blood was obtained following an over-night fast. Serum concentrations of lipids and glucose were performed on automatic biochemistry analyzer (Beckman Coulter AU5821, Brea, CA). Serum blood lipid levels were measured as previously described (19). Briefly, fasting serum TG and TC concentrations were determined using the enzymatic methods. LDL-C and HDL-C were measured using a direct homogeneous method. The inter-assay laboratory coefficient of variation (CV) of blood lipid testing ranged from 1.06% to 2.71%. Fasting blood glucose (FBG) concentration was detected by glucose oxidase method with CV of 1.25% at mean concentration of 3.27mmol/L. Fasting insulin (FINS) concentration was determined by the electro-chemiluminescence immunoassay (ECLI) method (CV 1.38%) on the full-automatic chemiluminescence immunoassay analyzer (cobas6000 e601, Roche Diagnostics, Germany) in the laboratory of the Department of Endocrinology and Metabolism. HOMA-IR was assessed by formula: HOMA-IR = FBG (mmol/L) x FINS (mIU/L)/22.5.

Reproductive hormone measurements were obtained at baseline coinciding with days 2-5 of the menstrual cycle or on the day of human chorionic gonadotrophin (HCG) triggering. FSH, LH, estradiol (E2), testosterone (T) and progesterone (P4) were quantified by ECLI in the laboratory at our center.

The controlled ovarian hyperstimulation (COH) protocols were individualized according to the age and ovarian reserve of infertile women as previously described (22). For the short-acting GnRH agonist protocol, subcutaneously injected 0.1mg triptorelin was scheduled for patients from the 7th day after ovulation to the 14th day after ovulation until sufficient downregulation of the pituitary was achieved. After that, exogenous gonadotropin (Gn) and 0.05mg triptorelin was administered simultaneously until the day of human chorionic gonadotropin (HCG) triggering. For the short protocol, 0.1mg triptorelin was administered every day from the day 2 of menstrual cycle until the day of HCG triggering. For the long-acting GnRH agonist protocol, patients received a single dose of leuprolide acetate (Enantone; 3.75mg) on day 2 of the menstrual cycle. If downregulation of the pituitary was satisfactory after 30 days, exogenous Gn was injected to initiate the cycle. In the ultra-long protocol, 3.75 mg of leuprolide acetate was repeatedly given after 28 days of the first dose administration, and Gn was injected 21 days after that. For the GnRH antagonist protocol, the Gn was injected on the 2-3 days of the cycle, and 0.25 mg of GnRH antagonist (Cetrotide) was supplied when the dominant follicle reached 12-14 mm in diameter and serum E2 was >150-400pg/ml until the day of HCG triggering.

Exogenous highly purified FSH (Lishenbao) and/or human menopausal gonadtrophin (HMG) (Lebaode) was injected to induce follicular development. Mainly based on age, BMI, and ovarian reserve, the initial dose of Gn ranged from 112.5-300.0IU/day and adjusted every 3-4 days according to ovarian response. When two or more follicles had reached a mean diameter of 18mm and the average E2 per mature follicle was 200-300pg/ml, 6000-10,000IU HCG was injected to promote the final maturation of the follicles. Oocytes were retrieved 36h after HCG administration, and this was followed by conventional IVF or ICSI.

At least one good quality cleavage-stage embryos were transferred on 3 days after follicle aspiration. Cleavage-stage embryos were evaluated according to ASEBIR embryo assessment criteria (23). All patients received oral progesterone capsules (Qining, 200mg/day) and vaginal micronized progesterone (Utrogestan, 600mg/day) for luteal support.

### Pregnancy Outcome and Follow-Up

Serum β-HCG concentration was measured 12 days after ET. Transvaginal B-ultrasound was performed on 28 and 35 days after ET to confirm an intrauterine pregnancy. After that, we would call the couple regularly to inquire about and record pregnancy and neonatal outcomes.

Clinical pregnancy was defined as observation of the gestational sac(s) in the uterine cavity by vaginal ultrasound on 4-5 weeks after ET. Clinical pregnancy rate was calculated by the ratio of clinical pregnancy cycle to the total ET cycle. Pregnancy that only detects β-hCG positivity in serum without seeing the gestational sac was termed as biochemical pregnancy. The implantation rate was defined as the number of gestational sacs divided by the number of transferred embryos. Early miscarriage was referred to intrauterine pregnancy loss before 12 weeks of pregnancy, while late miscarriage was referred to pregnancy loss prior to 28 weeks of gestational age. Preterm birth and term birth were defined as live birth before or after 37 gestational weeks. Low birth weight and macrosomia were defined as birth weight less than 2500g or more than 4000g, respectively (24).

### Statistical Analysis

Statistical analysis was carried out using SPSS (version 25). For continuous variables, if data satisfied a normal distribution, it was expressed as mean ± standard deviation, otherwise it was represented by the median and interquartile range (IQR). Categorical variables were presented as frequency and percentage. The between-group differences among variables were analyzed by one-way analysis of variance (ANOVA) or Kruskal-Wallis test, Pearson’s chi-squared test, or Fisher’s exact test for continuous and categorical variables, respectively. The Bonferroni method was used to correct P values for pairwise comparisons. The multivariable logistic regression model was adjusted for age, BMI, main aetiology of infertility, TG, LDL-C, HDL-C, antral follicle count (AFC), and endometrial type to demonstrate the association between HOMA-IR and late miscarriage rate. The associations were presented as an adjusted OR with a 95% confidence interval (CI). The P value was two-sided and value < 0.05 indicated statistical significance.

## Results

### Demographic and Clinical Characteristics of Participants According to the Quartiles of HOMA-IR

A total of 3,615 patients were enrolled in this study. The distribution of HOMA-IR was shown in **Supplementary Table 1**. The median of HOMA-IR was 1.99 with an IQR of 1.46-2.71. All subjects were divided into three groups according to the quartiles of HOMA-IR: Group 1 (HOMA-IR <1.46), Group 2 (HOMA-IR 1.46 to <2.71) and Group 3 (HOMA-IR ≥2.71).

The demographic and clinical characteristics of the 3,615 participants are presented in **Table 1**. Specifically, histories of previous therapeutic abortions, early spontaneous miscarriages, late miscarriages, and ectopic pregnancy did not differ among the three groups (P>0.05). BMI, serum levels of TG, LDL-C, glycated hemoglobin (HbA1c), FBG, FINS and basal T, and HOMA-IR increased with the HOMA-IR group (all P<0.001). Age, HDL-C level, and baseline hormone levels, including FSH, LH and E2 decreased with the HOMA-IR group (all P<0.05). Main aetiology of infertility and AFC significantly differed among the three groups and the percentage of ovulation disorder and AFC≥24 were highest in the Group 3 (all P<0.001).

**Table 1:** Demographic and clinical characteristics of participants according to the quartiles of HOMA-IR.

Parameter	Groups of cycles according to the quartiles of HOMA-IR			P-Value
	Group1 (<1.46) N=901	Group2 (1.46 to <2.71) N=1813	Group3 (≥2.71) N=901	
Age (years)	30.45 ± 4.18	30.05 ± 4.13	29.60 ± 4.26^ab^	<0.001
BMI (kg/m^2^)	20.14 ± 2.09	21.23 ± 2.48^a^	23.01 ± 2.88^ab^	<0.001
Infertility duration (years)	3.81 ± 2.87	3.72 ± 2.82	3.81 ± 2.72	0.640
Gravidity, n (%)				0.051
0	423 (46.95%)	865 (47.71%)	478 (53.05%)^ab^	
1	237 (26.30%)	475 (26.20%)	222 (24.64%)	
≥2	241 (26.75%)	473 (26.09%)	201 (22.31%)	
Parity, n (%)				0.506
0	712 (79.02%)	1460 (80.53%)	738 (81.91%)	
1	171 (18.98%)	327 (18.04%)	151 (16.76%)	
≥2	18 (2.00%)	26 (1.43%)	12 (1.33%)	
Previous therapeutic abortions, n (%)				0.171
0	659(73.14%)	1320(72.81%)	676(75.03%)	
1	161(17.87%)	339(18.70%)	170(18.87%)	
≥2	81(8.99%)	154(8.49%)	55(9.15%)	
Previous early spontaneous miscarriages, n (%)				0.352
0	833(92.45%)	1634(90.13%)	822(91.23%)	
1	60(6.66%)	153(8.44%)	67(7.44%)	
≥2	8(0.89%)	26(1.43%)	12(1.33%)	
Previous late miscarriages, n(%)				0.794
0	885(98.22%)	1786(98.51%)	885(98.22%)	
≥1	16(1.78%)	27(1.49%)	16(1.78%)	
Previous ectopic pregnancy,n(%)				0.06
0	738(81.91%)	1496(82.52%)	779(86.46%)	
1	117(12.99%)	234(12.91%)	86(9.54%)	
≥2	46(5.11%)	83(4.58%)	36(4.00%)	
Main aetiology of infertility, n (%)				<0.001
Tubal	660 (73.25%)	1289 (71.10%)	573 (63.60%)^ab^	
Ovulation disorder	69 (7.66%)	160 (8.83%)	136 (15.09%)^ab^	
Endometriosis	53 (5.88%)	110 (6.07%)	64 (7.10%)	
DOR	36 (4.00%)	60 (3.81%)	28 (3.11%)	
Male	80 (8.88%)	183 (10.09%)	97 (10.77%)	
Unexplained	3 (0.33%)	2 (0.10%)	3 (0.33%)	
TG (mmol/l)	0.82 ± 0.27	0.93 ± 0.30^a^	1.06 ± 0.31^ab^	<0.001
TC (mmol/l)	4.28 ± 0.54	4.33 ± 0.51	4.33 ± 0.03	0.051
LDL-C (mmol/l)	2.35 ± 0.47	2.42 ± 0.47^a^	2.49 ± 0.46^ab^	<0.001
HDL-C (mmol/l)	1.56 ± 0.31	1.50 ± 0.30^a^	1.41 ± 0.28^ab^	<0.001
HbA1c (%)	5.20 (5.00, 5.30)	5.20 (5.00, 5.40)^a^	5.30 (5.10, 5.50)^ab^	<0.001
FBG (mmol/L)	5.04 ± 0.37	5.25 ± 0.36^a^	5.47 ± 0.40^ab^	<0.001
FINS (uU/ml)	4.97 ± 1.09	8.66 ± 1.51^a^	15.44 ± 4.55^ab^	<0.001
HOMA-IR	1.11 ± 0.25	2.02 ± 0.35^a^	3.75 ± 1.16^ab^	<0.001
Basal FSH (mIU/ml)	6.74 (5.72, 8.20)	6.50 (5.40, 7.79)^a^	6.39 (5.36, 7.49)^a^	<0.001
Basal LH (mIU/ml)	5.26 (3.84, 6.80)	5.03 (3.59, 6.67)	4.80 (3.41, 6.68)^a^	0.022
Basal E2 (pg/ml)	38.46 (28.82, 49.50)	35.06 (25.56, 47.50)^a^	31.99 (22.33, 43.53)^ab^	<0.001
Basal T (ng/ml)	0.21 (0.14, 0.29)	0.22 (0.15, 0.31)^a^	0.26 (0.17, 0.35) ^ab^	<0.001
AFC, n (%)				<0.001
1-6	115 (12.76%)	190 (10.48%)	88 (9.77%)	
7-12	346 (38.40%)	683 (37.67%)	296 (32.85%)^ab^	
13-24	315 (34.96%)	637 (35.14%)	312 (34.63%)	
≥24	125 (13.88%)	303 (16.71%)	205 (22.75%)^ab^	
Ovarian stimulation protocol, n (%)				0.096
Short-acting GnRH agonist long protocol	405 (44.95%)	783 (43.19%)	374 (41.51%)	
Long-acting GnRH agonist long protocol	151 (16.76%)	382 (21.01%)	166 (18.42%)	
Ultra-long protocol	51 (5.66%)	116 (6.40%)	56 (6.22%)	
GnRH antagonist protocol	149 (16.54%)	254 (14.01%)	145 (16.09%)	
Short protocol	145 (16.09%)	278 (15.33%)	160 (17.76%)	
Duration of stimulation (days)	10.39 ± 2.17	10.61 ± 2.24	10.91 ± 2.58^ab^	0.006
Total Gn dose (IU)	2014.03 ± 739.53	2035.02 ± 703.91	2114.00 ± 719.33^ab^	<0.001
E2 on HCG Day (pg/ml)	2655.50(1797.25,3717.27)	2561.00(1692.00,3579.00)	2183.00(1486.00,3182.00)^ab^	<0.001
LH on HCG Day (mIU/ml)	1.70 (1.02, 2.72)	1.60 (0.89, 2.50)^a^	1.49 (0.88, 2.47)^a^	<0.001
P4 on HCG Day (ng/ml)	0.68 (0.47, 0.95)	0.70 (0.49, 0.93)	0.67 (0.48, 0.90)^ab^	0.574
Endometrial thickness (mm)	11.02 ± 2.21	11.11 ± 2.24	10.96 ± 2.06	0.232
Endometrial type, n (%)				<0.027
A	428 (47.50%)	880 (48.54%)	386 (42.84%)^b^	
B	436 (48.39%)	837 (46.17%)	458 (50.83%)	
C	37 (4.11%)	96 (5.29%)	57 (6.33%)	
No. of oocytes retrieved (n)	10.64 ± 4.42	10.99 ± 4.48	10.46 ± 4.28^b^	0.008
Fertilization method, n (%)				0.396
IVF	708 (78.58%)	1422 (78.43%)	687 (76.25%)	
ICSI	138 (15.32%)	266 (14.67%)	157 (17.43%)	
IVF+ICSI	55 (6.10%)	125 (6.89%)	57 (6.32%)	
No. of MII oocytes (n)	8.10 ± 3.95	8.41 ± 4.11	8.10 ± 3.51	0.100
MII oocyte rate (%)	7295/9588 (76.08%)	15256/19922 (75.58%)	7294/9420 (77.43%)	0.083
No. of good-quality embryos (n)	3.40 ± 2.20	3.48 ± 2.14	3.28 ± 1.91	0.245
Good-quality embryo rate (%)	3060/6143 (49.81%)	6301/13068 (48.22%)	2958/6120 (48.33%)	0.103
No. of embryos transferred (n)	1.84 ± 0.37	1.85 ± 0.36	1.87 ± 0.34	0.178
No. of good-quality embryos transferred (n)	1.60 ± 0.66	1.61 ± 0.67	1.64 ± 0.66	0.200
Good-quality embryos transferred rate, n (%)	1446/1654 (87.42%)	2910/3357 (86.68%)	1479/1682 (87.93%)	0.433

a P < 0.05, vs. Group 1

b P < 0.05, vs. Group 2;HOMA-IR, Homeostatic Model Assessment for Insulin Resistance; BMI, Body Mass Index; DOR, Diminished Ovarian Reserve; TG, serum Triglyceride; TC, Total Cholesterol; LDL-C, Low-Density Lipoprotein Cholesterol; HDL-C, High-Density Lipoprotein Cholesterol;HbA1c, Hlycated hemoglobin; FBG, Fasting Blood Glucose; FINS, Fasting Insulin; FSH, Follicle Stimulating Hormone; LH, Luteinizing Hormone;E2, Estradiol; T, Testosterone; AFC, Antral Follicle Count; GnRH, Gonadotropin Releasing Hormone; Gn, Gonadotropin; HCG, human chorionic gonadotropin; P4, Progesterone; IVF, In Vitro Fertilization; ICSI, Intracytoplasmic Sperm Injection; MII, Metaphase II.

With respect to clinical characteristics during the IVF/ICSI-ET procedure, duration of stimulation, total Gn dose, and the percentage of Type C endometrium on day of HCG triggering increased with the HOMA-IR group (all P<0.05). Serum E2 and LH levels on HCG administration day decreased with the HOMA-IR group (all P<0.001). The highest number of oocytes retrieved was observed in the Group 2, and the number of Group 1 was higher than that of Group 3 (P=0.008). Ovarian stimulation protocol, P4 level and endometrial thickness on day of HCG triggering, fertilization method, number of MII oocyte, MII oocyte rate, number of good-quality embryos, good-quality embryos rate, number of transferred embryos, number of good-quality embryos transferred, and good-quality embryos transferred rate exhibited no significant difference among the three groups (all P>0.05).

### Pregnancy and Neonatal Outcomes Among the Three Groups

**Figure 1:**
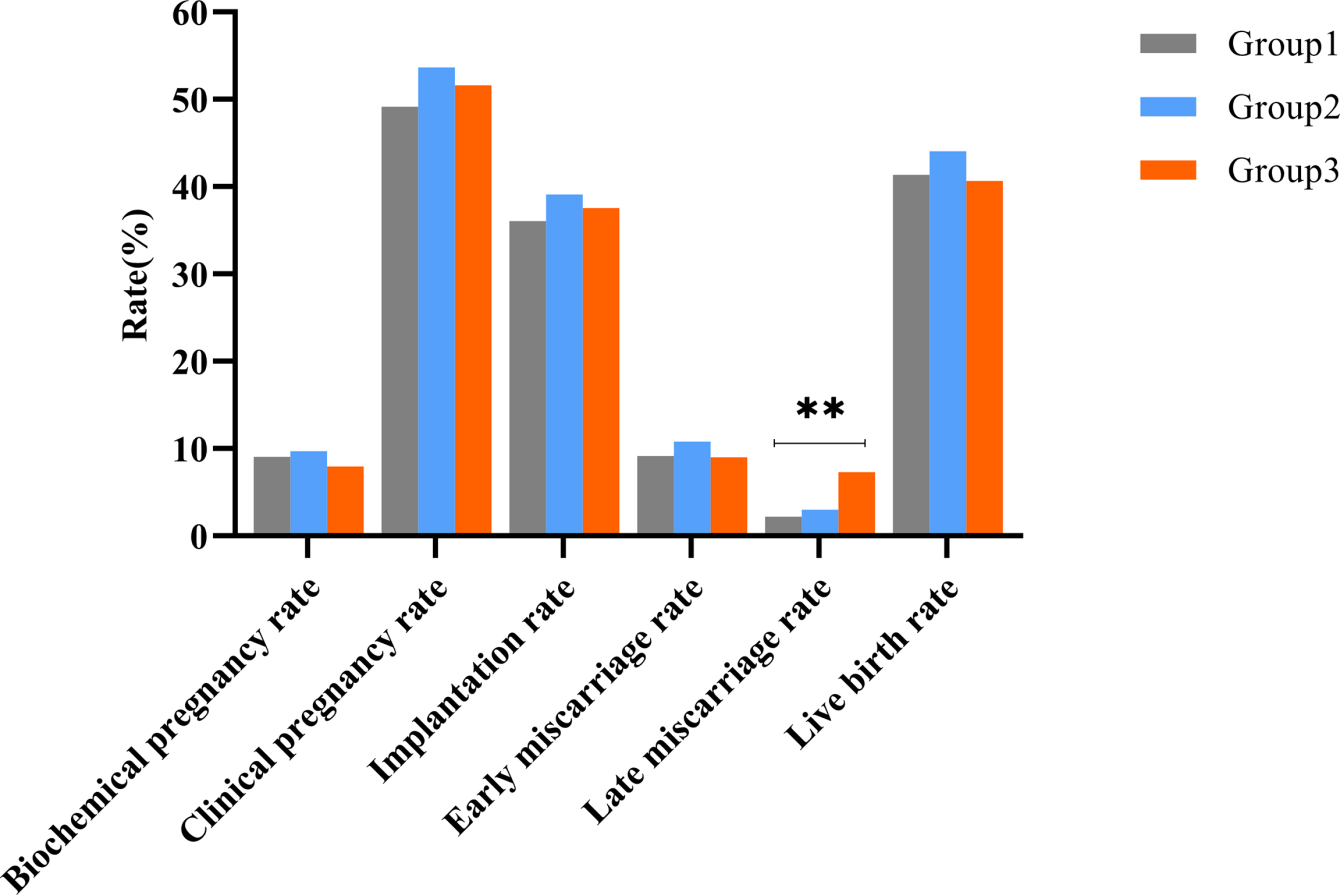
Clinical outcomes among the three groups during the first complete IVF/ICS-ET cycle. **Represented the differences among three groups were statistically significant.

Pregnancy and neonatal outcomes are illustrated in **Table 2** and are depicted in **Figure 1**. The late miscarriage rate and prevalence of macrosomia increased with the HOMA-IR group (for late miscarriage rate: 2.23% vs. 3.04% vs. 7.35%, P<0.001; for macrosomia: 0.21% vs. 1.70% vs. 3.23%, P=0.002). However, rates of biochemical pregnancy, clinical pregnancy, implantation, ectopic pregnancy, early miscarriage, preterm birth, term birth, live birth, and multiple pregnancy were comparable among the three groups (all P>0.05). Reasons for late miscarriage, percentage of low-birth weight infants, and gestational weeks did not differ among these groups as well (all P>0.05).

**Table 2:** Pregnancy and neonatal outcomes of participants according to the quartiles of HOMA-IR.

	Groups of cycles according to the quartiles of HOMA-IR				P-value
	All participants N=3615	Group1 (<1.46) N=901	Group2 (1.46 to <2.71) N=1813	Group3 (≥2.71) N=901	
Biochemical pregnancy rate	330/3615 (9.13%)	82/901 (9.10%)	176/1813 (9.71%)	72/901 (7.99%)	0.343
Clinical pregnancy rate	1881/3615 (52.03%)	443/901 (49.17%)	973/1813 (53.67%)	465/901 (51.61%)	0.083
Implantation rate	2543/6693 (37.99%)	597/1654 (36.09%)	1314/3357 (39.14%)	632/1682 (37.57%)	0.103
Ectopic pregnancy rate	63/3615 (1.74%)	19/901 (2.11%)	32/1813 (1.77%)	12/901 (1.33%)	0.460
Early miscarriage rate	171/1714 (9.98%)	37/403 (9.18%)	96/889 (10.80%)	38/422 (9.00%)	0.497
Late miscarriage rate	67/1714 (3.91%)	9/403 (2.23%)	27/889 (3.04%)	31/422 (7.35%)^ab^	<0.001
Reasons for late miscarriage, n (%)					0.383
Cervical insufficiency	4/67 (5.97%)	1/9 (11.11%)	1/27 (3.70%)	2/31 (6.45%)	
Intrauterine fetal death/Fetal malformation	48/67 (71.64%)	5/9 (55.56%)	22/27 (81.48%)	21/31 (67.74%)	
Premature rupture of the membrane	7/67 (10.45%)	1/9 (11.11%)	3/27 (11.11%)	3/31 (9.68%)	
Intrauterine infections	1/67 (1.49%)	0/9 (0.00%)	0/27 (0.00%)	1/31 (3.23%)	
Placenta previa	3/67 (4.48%)	0/9 (0.00%)	0/27 (0.00%)	3/31 (9.67%)	
External causes	4/67 (5.97%)	2/9 (22.22%)	1/27 (3.70%)	1/31 (3.23%)	
Preterm birth rate	258/3448 (7.48%)	67/861 (7.78%)	124/1729 (7.17%)	67/858 (7.81%)	0.785
Term birth rate	1179/3448 (34.19%)	289/861 (33.57%)	638/1729 (36.90%)	282/858 (32.87%)	0.073
Live birth rate	1467/3448 (42.55%)	356/861 (41.35%)	762/1729 (44.07%)	349/858 (40.68%)	0.174
Macrosomia,n(%)	33/1929 (1.71%)	1/466 (0.21%)	17/999 (1.70%)^a^	15/464 (3.23%)^a^	0.002
Low birth weight infants,n(%)	496/1929 (25.71%)	124/466 (26.61%)	260/999 (26.03%)	112/464 (24.14%)	0.654
Gestational weeks (w)	38 (37, 39)	38 (37, 39)	38 (37, 39)	38 (37, 39)	0.212
Multiple pregnancy rate	457/3448 (13.25%)	111/861 (12.89%)	233/1729 (13.48%)	113/858(13.17%)	0.144

a P < 0.05, vs. Group 1.

b P < 0.05, vs.Group 2;HOMA-IR, Homeostatic Model Assessment for Insulin Resistance.

Therefore, we further evaluated the association between HOMA-IR and late miscarriage (**Table 3**). Univariate logistic regression model indicated that women with increased HOMA-IR (≥2.71) were much likely to have a late miscarriage followed the fresh ET (crude OR 3.50, 95% CI 1.64-7.47, P=0.001) compared with the referent group (HOMA-IR<1.46). After adjusting for potential confounders, including age, BMI, main aetiology of infertility, TG level, LDL-C level, HDL-C level, AFC, and endometrial type in the multivariable logistic regression model, HOMA-IR≥2.71 remained a risk factor for late miscarriage (adjusted OR 3.56, 95%CI 1.56-8.15, P=0.003).

**Table 3:** Univariate and multivariate logistic regression analysis of the association between HOMA-IR and late miscarriage.

Variable	Univariate Binary Logistic Regression		Multivariate Logistic Regression	
	Crude OR (95% CI)	*P *value	Adjusted OR (95% CI)	*P *value
HOMA-IR group				
Group1 (<1.46)	Reference	–	Reference	–
Group2 (1.46 to < 2.71)	1.40 (0.65-3.00)	0.391	1.39 (0.63-3.04)	0.411
Group3 (≥2.71)	3.50 (1.64-7.47)	0.001	3.56 (1.56-8.15)	0.003

HOMA-IR, Homeostatic Model Assessment for Insulin Resistance; OR, Odds Ratio.

### Subgroup Analysis Based on Presence or Absence of PCOS

To evaluate the effects of HOMA-IR among non-PCOS populations, we performed a subgroup analysis based on presence or absence of the PCOS status (**Table 4**). According to the Rotterdam criteria (25), of the 3,615 participants, 450 were diagnosed with PCOS (12.45%). Among non-PCOS populations, BMI, serum levels of TG, LDL-C and basal T, HbA1c, duration of stimulation and total Gn dose increased with the HOMA-IR group (all P<0.001). Age, HDL-C level, and baseline hormone levels, including FSH, LH and E2 decreased with the HOMA-IR group (all P<0.01). Also, types of infertility, main aetiology of infertility, endometrial type, and number of oocytes retrieved differed significantly among the three groups (all P<0.05). The late miscarriage rate was highest in the Group 3, which was consistent with the overall population results (P<0.001).

**Table 4:** The baseline, clinical characteristics and pregnancy outcomes of patients with or without PCOS.

Variables	Non-PCOS (n=3165)					PCOS (n=450)			
	Group1 (<1.46) N=805		Group2 (1.46 to <2.71) N=1612	Group3 (≥2.71)N=748	P value	Group1 (<1.46) N=96	Group2 (1.46 to <2.71) N=201	Group3 (≥2.71) N=153	P value
Age (years)	30.69 ± 4.12	30.30 ± 4.14		29.95 ± 1.30^a^	0.002	28.45 ± 4.11	27.99 ± 3.32	27.89 ± 3.60	0.638
BMI (kg/m^2^)	20.11 ± 2.07	21.19 ± 2.49^a^		22.84 ± 2.86^ab^	<0.001	20.35 ± 2.26	21.49 ± 2.43^a^	23.91 ± 2.83^ab^	<0.001
Type of infertility n (%)					0.021				0.177
Primary	355 (44.10%)	745 (46.22%)		381 (50.94%)^a^		68 (70.83%)	120 (59.70%)	97 (63.40%)	
Secondary	450 (55.90%)	867 (53.78%)		367 (49.06%)		28 (29.17%)	81 (40.30%)	56 (36.60%)	
Main aetiology of infertility n (%)					0.023				0.261
Tubal	629 (78.14%)	1234 (76.55%)		540 (72.19%)^a^		31 (32.29%)	55 (27.36%)	33 (21.57%)	
Ovulatory	11 (1.37%)	37 (2.30%)		31 (4.14%)^ab^		58 (60.42%)	123 (61.19%)	105 (68.63%)	
Endometriosis	53 (6.58%)	106 (6.58%)		61 (8.16%)		0 (0.00%)	4 (1.99%)	3 (1.96%)	
DOR	36 (4.47%)	69 (4.28%)		28 (3.74%)		0 (0.00%)	0 (0.00%)	0 (0.00%)	
Male	74 (9.19%)	164 (10.17%)		85 (11.36%)		6 (6.25%)	19 (9.45%)	12 (7.84%)	
Unexplained	2 (0.25%)	2 (0.12%)		3 (0.40%)		1 (1.04%)	0 (0.00%)	0 (0.00%)	
TG (mmol/l)	0.82 ± 0.27	0.92 ± 0.30^a^		1.05 ± 0.32^ab^	<0.001	0.86 ± 0.26	0.98 ± 0.30^a^	1.10 ± 0.29^ab^	<0.001
TC (mmol/l)	4.27 ± 0.54	4.33 ± 0.51		4.32 ± 0.53	0.100	4.30 ± 0.48	4.35 ± 0.50	4.35 ± 0.55	0.505
LDL-C (mmol/l)	2.34 ± 0.48	2.42 ± 0.47^a^		2.47 ± 0.46^ab^	<0.001	2.44 ± 0.43	2.47 ± 0.46	2.58 ± 0.46^a^	0.023
HDL-C (mmol/l)	1.57 ± 0.31	1.51 ± 0.30^a^		1.42 ± 0.29^ab^	<0.001	1.51 ± 0.30	1.49 ± 0.28	1.36 ± 0.28^ab^	<0.001
HbA1c (%)	5.20 (5.00, 5.30)	5.20 (5.00, 5.40)^a^		5.30 (5.10, 5.50)^ab^	<0.001	5.20 (5.08, 5.40)	5.20 (5.10, 5.40)	5.30 (5.10, 5.50)^b^	0.025
Basal FSH (mIU/ml)	6.80 (5.80, 8.26)	6.59 (5.50, 7.82)^a^		6.43 (5.50, 7.59)^a^	<0.001	6.01 (5.36, 7.35)	5.80 (4.60, 7.02)	5.80 (4.88, 6.90)	0.050
Basal LH (mIU/ml)	5.09 (3.73, 6.50)	4.91 (3.52, 6.46)		4.57 (3.35, 6.07)^ab^	<0.001	7.31 (5.07, 9.68)	7.09 (4.41, 12.05)	7.03 (4.70, 12.14)	0.901
Basal E2 (pg/ml)	38.68(28.56,49.56)	34.86(25.37,47.30)^a^		31.11(21.51,42.69)^ab^	<0.001	37.75(32.65,46.70)	36.16(26.82,51.02)	36.90(28.10,46.43)	0.543
Basal T (ng/ml)	0.20 (0.14, 0.29)	0.21 (0.15, 0.29)		0.24 (0.17, 0.32) ^ab^	<0.001	0.23 (0.19, 0.35)	0.32 (0.23, 0.49)^a^	0.36 (0.26, 0.48)^a^	<0.001
AFC n (%)					0.213				<0.001
1-6	115 (14.29%)	190 (11.79%)		88 (11.76%)		0 (0.00%)	0 (0.00%)	0 (0.00%)	
7-12	342 (42.48%)	678 (42.06%)		293 (39.17%)		4 (4.17%)	5 (2.49%)	3 (1.96%)	
13-24	276 (34.29%)	598 (37.10%)		285 (38.10%)		39 (40.63%)	39 (19.40%)^a^	27 (17.65%)^a^	
≥24	72 (8.94%)	146 (9.06%)		82 (10.96%)		53 (55.21%)	157 (78.11%)^a^	123 (80.39%)^a^	
Ovarian stimulation protocol n (%)					0.079				0.177
Short-acting GnRH agonist long protocol	367 (45.59%)	712 (44.17%)		306 (40.91%)		38 (39.58%)	71 (35.32%)	68 (44.44%)	
Long-acting GnRH agonist long protocol	130 (16.15%)	316 (19.60%)		127 (16.98%)		21 (21.88%)	66 (32.84%)	29 (25.49%)	
Ultra-long protocol	50 (6.21%)	112 (6.95%)		54 (7.22%)		1 (1.04%)	4 (1.99%)	2 (1.31%)	
GnRH antagonist protocol	127 (15.78%)	224 (13.90%)		114 (15.24%)		22 (22.92%)	30 (14.93%)	31 (20.26%)	
Short protocol	131 (16.27%)	248 (15.38%)		147 (19.65%)		14 (14.58%)	30 (14.92%)	13 (8.50%)	
Duration of stimulation (days)	10.38 ± 2.06	10.51 ± 2.12		10.71 ± 2.31^a^	0.035	10.50 ± 2.96	11.38 ± 2.94^a^	11.88 ± 3.47^a^	0.002
Total Gn dose (IU)	2074.00 ± 723.29	2074.63 ± 695.90		2147.58 ± 690.36^ab^	0.013	1511.22 ± 683.96	1717.36 ± 688.60^a^	1949.82 ± 829.96^ab^	<0.001
Endometrial thickness (mm)	11.05 ± 2.23	11.15 ± 2.25		11.06 ± 2.06	0.644	10.79 ± 1.98	10.81 ± 2.18	10.47 ± 1.96	0.281
Endometrial type n (%)					0.043				0.874
A	385 (47.83%)	796 (49.38%)		327 (43.72%)^b^		43 (44.74%)	84 (41.79%)	59 (38.56%)	
B	388 (48.20%)	734 (45.53%)		374 (50.00%)		48 (50.00%)	103 (51.24%)	84 (54.90%)	
C	32 (3.97%)	82 (5.09%)		47 (6.28%)		5 (5.21%)	14 (6.97%)	10 (6.54%)	
No. of oocytes retrieved n (%)	10.49 ± 4.43	10.88 ± 4.44		10.22 ± 4.29^b^	0.002	11.94 ± 4.13	11.88 ± 4.74	11.61 ± 4.23	0.821
Fertilization method n (%)					0.214				0.214
IVF	626 (77.76%)	1268 (78.66%)		562 (75.13%)		82 (85.42%)	154 (76.62%)	125 (81.70%)	
ICSI	126 (15.65%)	237 (14.70%)		139 (18.58%)		12 (12.50%)	29 (14.43%)	18 (11.76%)	
IVF+ICSI	53 (6.59%)	107 (6.64%)		47 (6.29%)		2 (2.08%)	18 (8.95%)	10 (6.54%)	
No. of MII oocyte (n)	7.96 ± 3.92	8.31 ± 4.09		7.94 ± 3.44	0.054	9.27 ± 4.03	9.23 ± 4.19	8.85 ± 3.78	0.618
Good-quality embryo rate	2708 (49.95%)	5548 (48.29%)^a^		2412 (48.45%)^b^	0.116	352 (48.75%)	753 (47.72%)	546 (47.81%)	0.892
No. of transferred embryos (n)	1.83 ± 0.37	1.86 ± 0.35		1.88 ± 0.33	0.070	1.84 ± 0.37	1.81 ± 0.40	1.82 ± 0.38	0.724
Clinical pregnancy rate	400/805 (49.69%)	868/1612 (53.85%)		388/748 (51.87%)	0.150	43/96 (44.79%)	105/201 (52.24%)	77/153 (50.33%)	0.484
Implantation rate	534/1477 (36.15%)	1173/2994 (39.18%)		530/1403 (37.78%)	0.142	63/177 (35.59%)	141/363 (38.84%)	102/279 (36.56%)	0.721
Early miscarriage rate	33/363 (9.09%)	90/791 (11.38%)		31/352 (8.81%)	0.297	4/40 (10.00%)	6/98 (6.12%)	7/70 (10.00%)	0.571
Late miscarriage rate	8/363 (2.20%)	24/791 (3.03%)		27/352 (7.67%)^ab^	<0.001	1/40 (2.50%)	3/98 (3.06%)	4/70 (5.71%)	0.634

a P < 0.05, vs. Group 1.

b P < 0.05, vs. Group 2; PCOS, Polycystic Ovary Syndrome; HOMA-IR, Homeostatic Model Assessment for Insulin Resistance; BMI, Body Mass Index; DOR, Diminished Ovarian Reserve; TG, serum Triglyceride; TC, Total Cholesterol; LDL-C, Low-Density Lipoprotein Cholesterol; HDL-C, High-Density Lipoprotein Cholesterol; HbA1c, Hlycated hemoglobin; FBG, Fasting Blood Glucose; FINS, Fasting Insulin; FSH, Follicle Stimulating Hormone; LH, Luteinizing Hormone; E2, Estradiol; T, Testosterone; AFC, Antral Follicle Count; GnRH, Gonadotropin Releasing Hormone; Gn, Gonadotropin; HCG, human chorionic gonadotropin; P4, Progesterone; IVF, In Vitro Fertilization; ICSI, Intracytoplasmic Sperm Injection; MII, Metaphase II.

In PCOS populations, BMI, serum levels of TG, LDL-C, HDL-C and basal T, HbA1c, AFC, duration of stimulation, and total Gn dose exhibit significant differences among the three groups (all P<0.05). However, pregnancy outcomes were comparable among the three groups (P>0.05).

Consequently, we evaluated the association between HOMA-IR and late miscarriage among non-PCOS populations by adopting univariate logistic regression and multivariable logistic regression models (**Supplementary Table 2**). Likewise, in the univariate logistic regression model, women with increased HOMA-IR (≥2.71) were much likely to have a late miscarriage (crude OR 3.71, 95% CI 1.66-8.30, P=0.001) compared with the referent group (HOMA-IR<1.46). After adjusting potential confounders, e.g., age, BMI, type of infertility, main aetiology of infertility, TG level, LDL-C level, HDL-C level, AFC and endometrial type, HOMA-IR≥2.71 remained a risk factor for late miscarriage (adjusted OR 3.82, 95%CI 1.59-9.17, P=0.003).

## Discussion

Maternal metabolism disturbances have long been linked to abnormalities in endometrial function and fetal development (26, 27). Previously, we demonstrated that women with dyslipidemia were less likely to have a live birth compared with the non-dyslipidemic women (19). To tease out the effects of IR from that of accompanying dyslipidemia, we enrolled non-dyslipidemic infertile women in the present study. It is suggested that higher HOMA-IR was positively associated with late miscarriage in normolipidemic women undergoing fresh ET.

Whether IR, per se, is associated with increased miscarriage is controversial. To date, there is limited data concerning to the relationship between IR and miscarriage after ART treatment, and majority of the data is confined to PCOS individuals. In recent meta-analysis, IR was demonstrated to be associated with an increased risk of spontaneous abortion, which was defined as a pregnancy loss before 20 weeks of pregnancy, in PCOS patients undergoing ART (15). A retrospective cohort study involving 2,231 PCOS patients indicated a higher level of HOMA-IR in the spontaneous abortion group than those in ongoing pregnancy group and HOMA-IR was closely related with spontaneous abortion occurrence (28). Tian et al.’s study revealed that even after adjusting for PCOS status, HOMA-IR still remained a risk factor for spontaneous miscarriage during fresh ET (16). Similarly, in another study with a smaller sample size, BMI, FINS, HOMA-IR and serum chemerin levels were positively correlated with the occurrence of abortion in PCOS women (29). Nevertheless, after adjusted for BMI in multivariable logistic regression, the association between HOMA-IR and the spontaneous abortion did not exist (29).

Other reports, however, dispute these results (17, 30, 31). Wang et al. failed to find significant differences in pregnancy outcomes, including pregnancy rate, clinical pregnancy loss rate, and cumulative live birth rate, between IR and non-IR group (17). Among non-PCOS participants, no associations were found between hyperinsulinemia and IR and clinical pregnancy, live birth, and miscarriage (31). A large randomized controlled trial involving PCOS women undergoing ovulation induction indicated that the rates of conception, clinical pregnancies, and live births were all significantly reduced with the increase in FINS or HOMA-IR level, while miscarriage rates were not related to FINS or HOMA-IR (32).

In the current study, late miscarriage rate was higher in Group 3 (HOMA-IR≥2.71) than those in Group 2 (HOMA-IR 1.46 to < 2.71) and Group 1 (HOMA-IR<1.46). After controlling of potential confounders, a higher level of HOMA-IR was a risk factor for late miscarriage. The subgroup analysis indicated that this conclusion was applicable to the non-PCOS women. We failed to distinguish significant difference between HOMA-IR and late miscarriage rate in women diagnosed with PCOS and this might be ascribed to the limited population number in our study. These controversial results may be attributed to multifaceted factors such as the heterogeneity in terms of study design, study populations, sample size, diagnostic criteria of IR, ovarian stimulation protocols, inclusion, and exclusion criteria.

Associations between IR and recurrent miscarriage or pregnancy loss in women who conceived naturally has been reported in previous studies (33–35). The mechanisms linking IR with the risk of miscarriage remain unclear. Hyperinsulinemia and IR would create excessive glucose transport to the fetal environment by upregulating the glucose transporters (36, 37). Women diagnosed with gestational diabetes mellitus (GDM) in the first trimester had a higher probability of late miscarriage (38). Receiving metformin, an insulin-sensitizing drug, the late in the first trimester until delivery, might reduce the risks of late miscarriage and preterm birth (39), implying the effects of dysregulated glucose and insulin metabolism on the pregnancy outcomes in the mid-trimester of pregnancy. Except for this, altered maternal glucose and insulin metabolism would impact endometrial function on the transcriptomic and proteomic levels (40). Mice in early-stage pregnancy exposed to high insulin levels markedly compromised the decidualization process by attenuating endometrial vascularization and inhibiting endometrial stromal cells apoptosis (11, 41). In the present study, we noted an increased prevalence of C type endometrium with the elevated trend of HOMA-IR level, suggesting a defective endometrial milieu among patients with IR.

To our current knowledge, there is no report regarding the relationship between preconception IR and late miscarriage among non-dyslipidemic populations. Consistent with a previous study (31, 32), our data shows an increased trend in serum levels of TG, LDL-C and a decreased trend in serum HDL-C level with the increased HOMA-IR group, even though the serum lipid levels were normal. After controlling for potential confounders, we confirmed that HOMA-IR is an independent risk factor for late miscarriage in either normolipidemic or non-PCOS women. Moreover, occurrence of macrosomia increased with the HOMA-IR group, indicating that a pregestational insulin signaling disturbance might be associated with the risk of macrosomia in neonates. Although the results are compelling, several drawbacks of this study should be acknowledged. One of the major limitations lies in its retrospective nature, especially the high potential heterogeneity and possible confounding factors among the study populations. As a tertiary hospital in Hunan province, some patients from other provinces in China would be transferred to the obstetrics department in their hometown after obtaining a clinical pregnancy in our center. In this case, we were not able to fully record some details about gestational complications and comorbidities during their pregnancy, e.g., premonitory abortion in early stage and GDM. Besides, due to its retrospective nature, the information about participants’ lifestyle in terms of physical activity and diet before ART is also not available. Second, although over 3,500 individuals were enrolled in this study, the relatively small sample size of the PCOS subgroup may have hindered the detection of significant differences among this population. Third, the present study only includes D3 cleavage embryo without preimplantation genetic testing for aneuploidy (PGT-A). We cannot rule out the embryonic effects on late miscarriage after IVF/ICSI-ET. In the future, a prospective clinical study with more detailed information about the lifestyle, more restrict inclusion criteria, and embryos transferred after PGT-A selection is needed to confirm the results. Finally, generalization of the study findings could be limited to some extent by selection bias due to the nature of the single-center analysis and population difference between Chinese and other races.

In conclusion, late miscarriage rate and prevalence of macrosomia increased with the HOMA-IR index. Preconception HOMA-IR is an independent risk factor for late miscarriage in normolipidemic women undergoing IVF/ICSI-ET. Control of insulin resistance before ART might prevent the occurrence of late miscarriage and macrosomia.

## Data Availability Statement

The original contributions presented in the study are included in the article/**Supplementary Material**, further inquiries can be directed to the corresponding authors.

## Ethics Statement

The studies involving human participants were reviewed and approved by Ethical Committee of Xiangya Hospital of Central South University (CSU) (2021008). Written informed consent for participation was not required for this study in accordance with the national legislation and the institutional requirements.

## Author Contributions

TY and YY were responsible for data collection, article review and draft of the manuscript. QZ, DL, and NL contributed to the data verification. YML and ZY performed the statistical analysis. ZY and FT were responsible for the manuscript revision and designs of tables and figure. JZ and YPL are corresponding authors and they critically revised the manuscript. All authors contributed to the article and approved the submitted version.

## Funding

This study was supported by the National Key Research and Development Program of China (2021YFC2700404) and the Key Project of Research and Development Plan in Hunan Province (2021SK2028).

## Conflict of Interest

The authors declare that the research was conducted in the absence of any commercial or financial relationships that could be construed as a potential conflict of interest.

## Publisher’s Note

All claims expressed in this article are solely those of the authors and do not necessarily represent those of their affiliated organizations, or those of the publisher, the editors and the reviewers. Any product that may be evaluated in this article, or claim that may be made by its manufacturer, is not guaranteed or endorsed by the publisher.
