# Supplementary material for: Homeostatic Model Assessment for Insulin Resistance Is Associated With Late Miscarriage in Non-Dyslipidemic Women Undergoing Fresh IVF/ICSI Embryo Transfer
Source: Front Endocrinol (Lausanne). 2022 Jun 17;13:880518. doi: 10.3389/fendo.2022.880518 (PMC9247267; doi:10.3389/fendo.2022.880518)
Supplement: Supplementary file 1 [file Table_1.docx]

Supplementary Table 1. Different percentiles of HOMA-IR.

|  | P5 | P10 | P25 | P50 | P75 | P90 | P95 |
| --- | --- | --- | --- | --- | --- | --- | --- |
| HOMA-IR | 0.89 | 1.09 | 1.46 | 1.99 | 2.71 | 3.61 | 4.36 |
| Note: HOMA-IR= Homeostatic Model Assessment for Insulin Resistance. | | | | | | | |
