# Supplementary material for: Homeostatic Model Assessment for Insulin Resistance Is Associated With Late Miscarriage in Non-Dyslipidemic Women Undergoing Fresh IVF/ICSI Embryo Transfer
Source: Front Endocrinol (Lausanne). 2022 Jun 17;13:880518. doi: 10.3389/fendo.2022.880518 (PMC9247267; doi:10.3389/fendo.2022.880518)
Supplement: Supplementary file 2 [file Table_2.docx]

| Supplementary Table2. Association between HOMA-IR and late miscarriage among non-PCOS participants. | | | | |
| --- | --- | --- | --- | --- |
| Variable | Univariate Binary Logistic Regression | | Multivariate Logistic Regression | |
|  | Crude OR (95% CI) | *P*value | Adjusted OR (95% CI) | *P*value |
| HOMA-IR |  |  |  |  |
| Group1(<1.46) | Reference |  | Reference |  |
| Group2(1.46 to <2.71) | 1.43 (0.63-3.21) | 0.391 | 1.41 (0.61-3.24) | 0.418 |
| Group3(≥2.71) | 3.71 (1.66-8.30) | 0.001 | 3.82 (1.59-9.17) | 0.003 |
| Note: HOMA-IR= Homeostatic Model Assessment for Insulin Resistance; OR=Odds Ratio. | | | | |
